# Supplementary material for: Nanosecond chain dynamics of single-stranded nucleic acids
Source: Nat Commun. 2024 Jul 17;15:6010. doi: 10.1038/s41467-024-50092-8 (PMC11255343; doi:10.1038/s41467-024-50092-8)
Supplement: Supplementary file 1 — Supplementary Information [file 41467_2024_50092_MOESM1_ESM.pdf]

Supplementary Information for

Nanosecond chain dynamics of single-stranded nucleic acids

Mark F. Nüesch[a], Lisa Pietrek[b], Erik D. Holmstrom[a,c,d*], Daniel Nettels[a], Valentin von Roten[a], Rafael Kronenberg-Tenga[a], Ohad Medalia[a], Gerhard Hummer[b,e*], Benjamin Schuler[a,f*]

[a] Department of Biochemistry, University of Zurich, Winterthurerstrasse 190, 8057 Zurich, Switzerland

[b] Department of Theoretical Biophysics, Max Planck Institute of Biophysics, Max-von-Laue-Straße 3, 60438 Frankfurt am Main, Germany

[c] Department of Chemistry, University of Kansas, Lawrence, KS, USA

[d] Department of Molecular Biosciences, University of Kansas, Lawrence, KS, USA

[e] Institute for Biophysics, Goethe University Frankfurt, 60438 Frankfurt am Main, Germany

[f] Department of Physics, University of Zurich, Winterthurerstrasse 190, 8057 Zurich, Switzerland

* To whom correspondence should be addressed

Email: erik.d.holmstrom@ku.edu, gerhard.hummer@biophys.mpg.de, schuler@bioc.uzh.ch

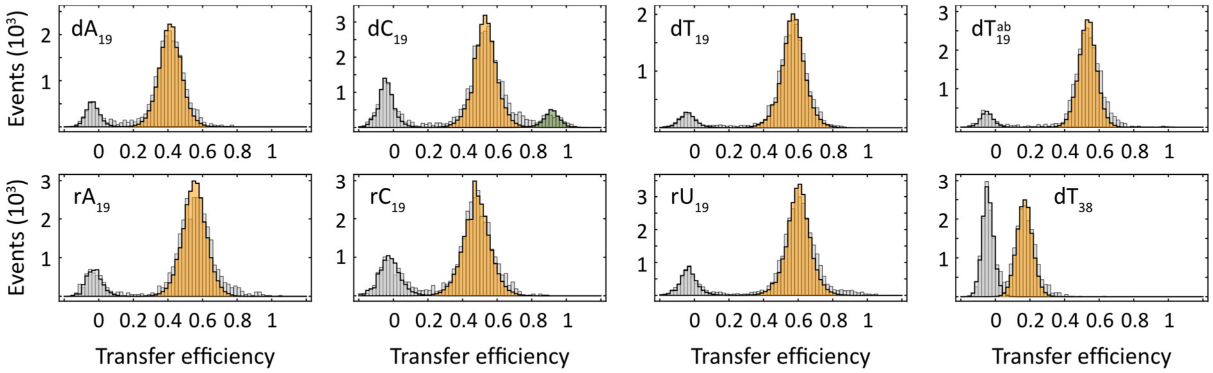

Supplementary Figure 1. Transfer efficiency histograms of ssNAs. Transfer efficiency histograms (gray) of ssNAs at 150 mM NaCl in 10 mM HEPES buffer pH 7 fitted with shot noise-limited photon distribution analysis (PDA)[1-3] (black solid line). The donor-only populations obtained from PDA ($E \approx 0$) are shown in dark gray and the FRET populations in orange. The similarity in width of measured and PDA histograms indicates the absence of dynamics on a timescale of the diffusion time (300 μs – 1 ms) or slower. dC_{19} shows a second FRET-active population (pale green), which was excluded from the lifetime and nsFCS analyses. This population increases at low salt, low pH, and low temperatures, and is likely to correspond to the formation of an i-motif[4]. Source data are provided as a Source Data file.

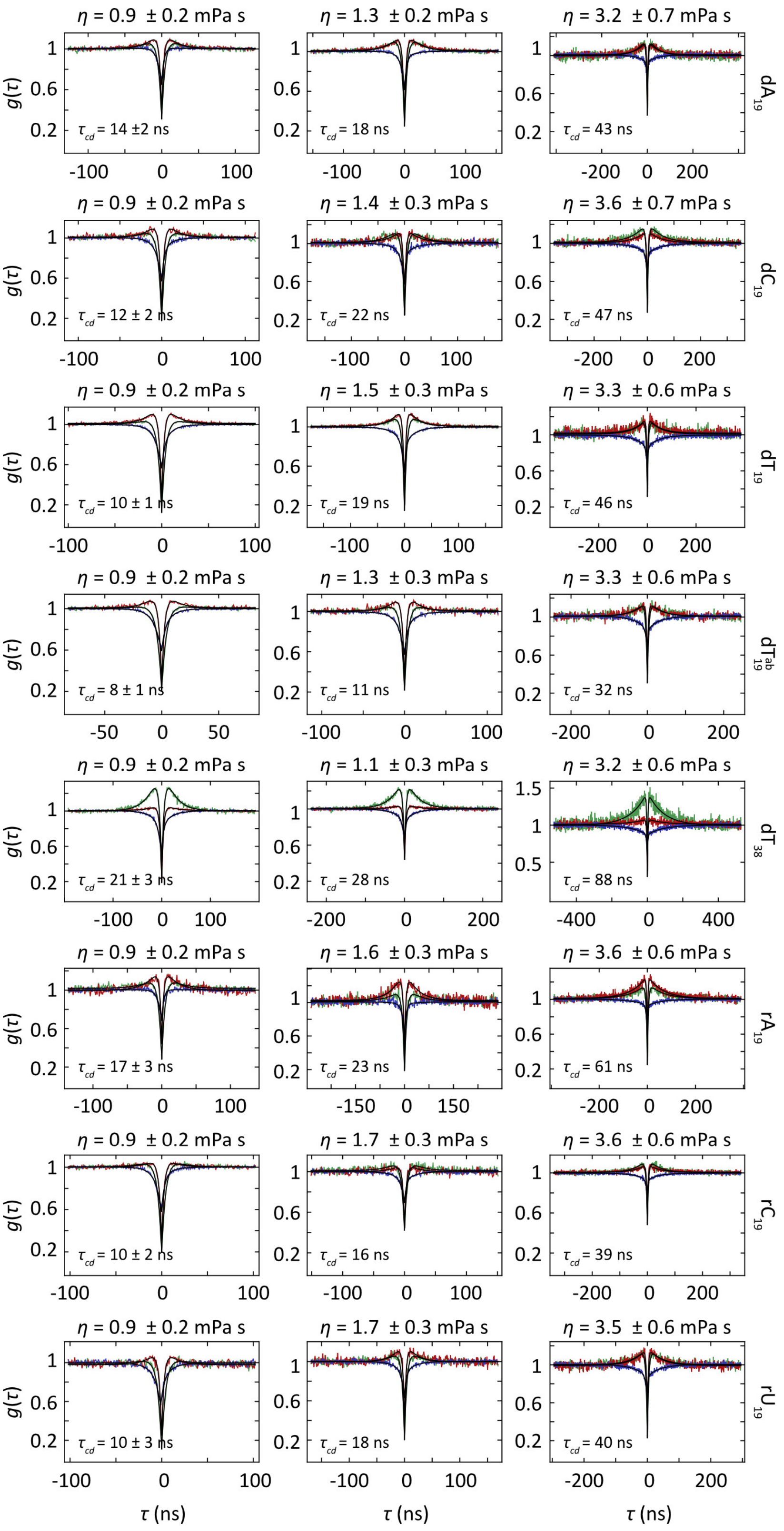

Supplementary Figure 2. Example data from nsFCS of ssNAs at different solvent viscosities. Normalized donor (green) and acceptor (red) fluorescence auto- and crosscorrelation (blue) curves of ssNAs at different solvent viscosities, η, with fits (Eq. 11, black solid lines) and timescale of chain dynamics (τ_{cd}) indicated. The uncertainty of τ_{cd} in the absence of viscogen (η = 0.9 ± 0.2 mPa s) represents the standard deviation from three measurements, two without and one with ZMW. See Methods for information on the uncertainty in solvent viscosity. The small negative amplitudes in the crosscorrelations measured for dA_{19} and rA_{19} indicate a low amplitude of long-range distance dynamics. Source data are provided as a Source Data file.

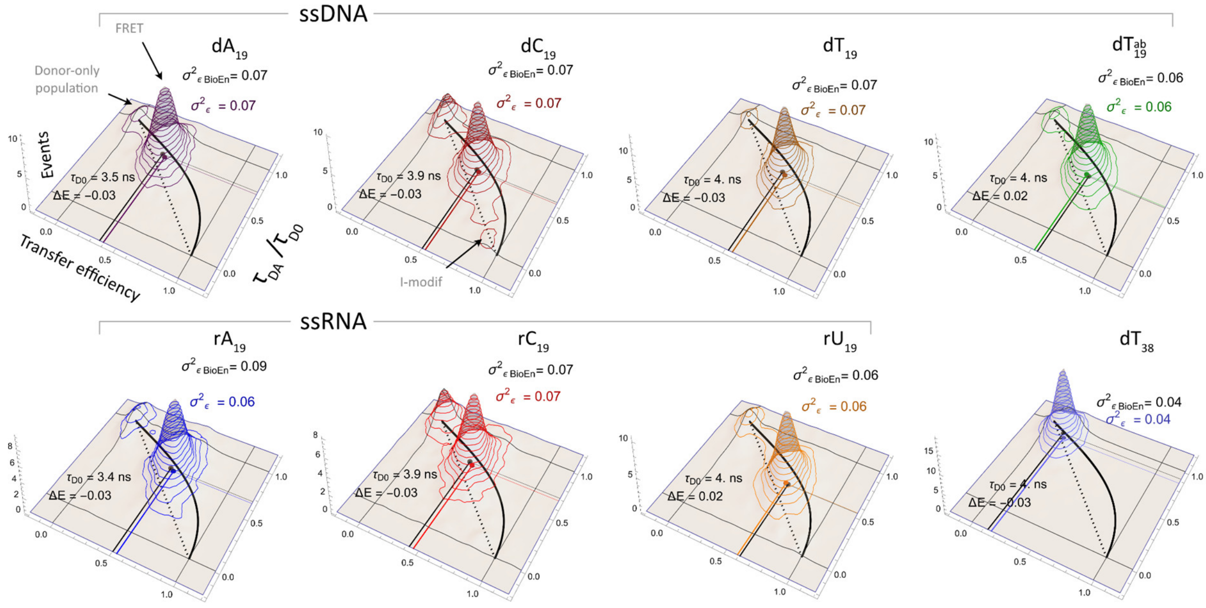

Supplementary Figure 3. 2D histograms of relative donor fluorescence lifetime versus transfer efficiency. Joint distributions of relative lifetime and transfer efficiency of ssNA variants measured at 150 mM NaCl in HEPES buffer pH 7. Indicated are the variances, σ_{ε}^{2} (colored) and $\sigma_{\varepsilon\ \mathrm{BioEn}}^{2}$ (black), donor lifetimes in the absence of FRET, τ_{D0} (see SI/Methods, Table S2), and differences of mean transfer efficiency, $\Delta E = \langle \varepsilon \rangle - \langle E \rangle$, between experimental values and values expected from the $\mathrm{HCG_{BioEn}}$ ensembles (for calculation of $\langle \varepsilon \rangle$ from $\mathrm{HCG_{BioEn}}$ ensembles, see *Bayesian ensemble refinement*). The black solid curves represent the dynamic lines calculated for the WLC polymer model by varying the persistence length (Eq. 2, Supplementary Table 3), and the black dashed lines represent the relation expected for static inter-dye distances. In the case of $\mathrm{dC_{19}}$, a second population at $E \approx 0.9$ is visible, which was excluded from further analysis. For $\mathrm{dT_{38}}$, the donor-only population was excluded for clarity by excluding photon burst with PIE stoichiometry ratios above 0.7. Source data are provided as a Source Data file.

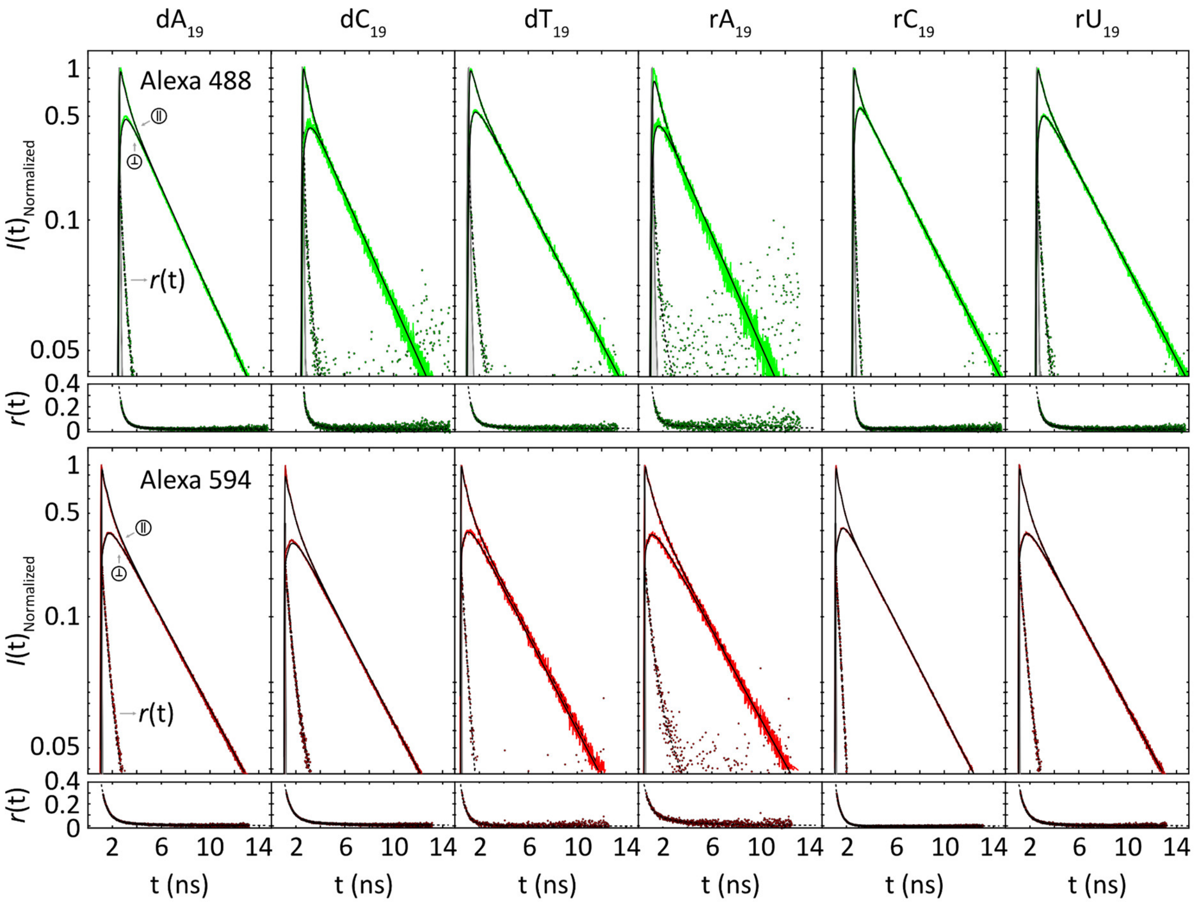

Supplementary Figure 4. Fluorescence lifetime and anisotropy decays of Alexa Fluor 488 and 594 conjugated to ssNAs. Normalized parallel (‖) and perpendicular (⊥) polarized fluorescence intensity, *I*(*t*), and anisotropy decays, *r*(t) (points), of single-donor labeled (top, green) and double-labeled (bottom, red) ssNAs (150 mM NaCl in HEPES buffer pH7) after donor (top) and acceptor excitation (bottom), respectively, with fits (solid and dashed black lines: Eq. 12, see Table S2) and instrument response function (gray). The rapid anisotropy decays of both donor and acceptor indicate high mobility of the fluorophores. Source data are provided as a Source Data file.

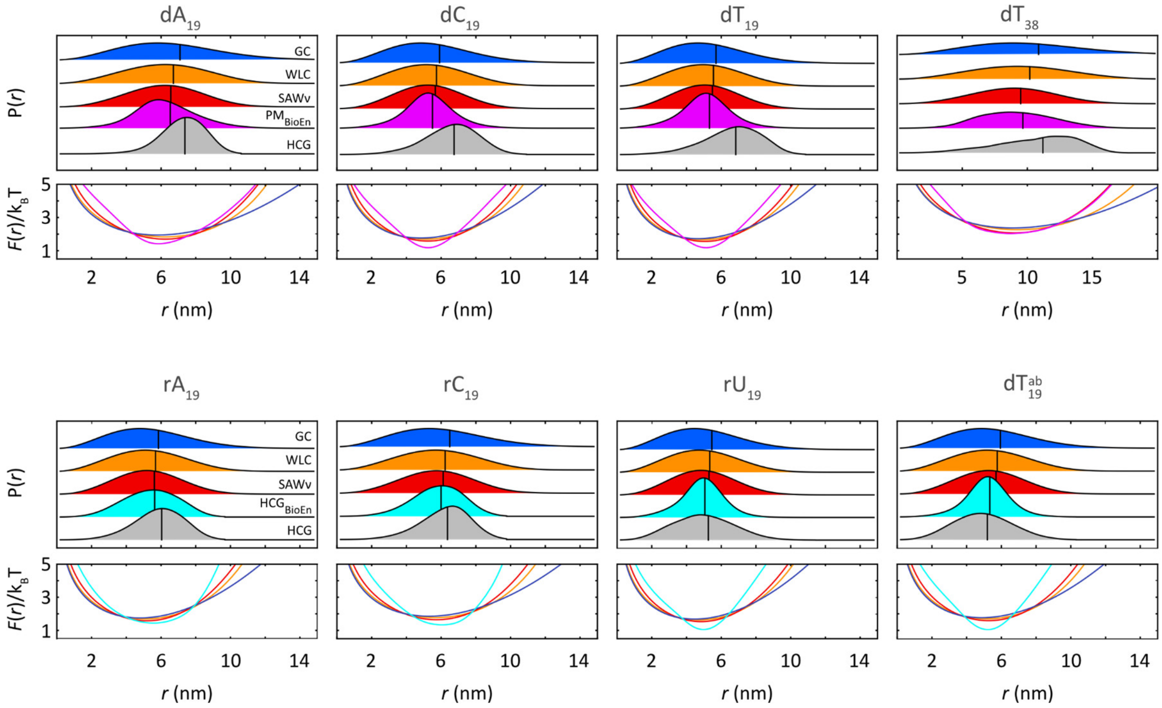

Supplementary Figure 5. Dye-to-dye distance distributions and corresponding potentials of mean force from polymer models and hierarchical chain growth. Probability density functions, *P*(*r*), of the dye-to-dye distances and corresponding potentials of mean force, *F*(*r*), from polymer models (GC: blue, WLC: orange, SAW-v: red) and from HCG (gray). The HCG ensembles for dA_{19}, dC_{19}, dT_{19}, and dT_{38} were not used in the analysis of the chain reconfiguration times and are only shown for reference (see *Bayesian ensemble refinement* for details). The reweighted distributions (PM_{BioEn}, HCG_{BioEn}) are shown in purple and cyan, respectively (reweighted according to the experimentally obtained means and variances of the transfer efficiency distributions of ssNAs, see Methods). Root mean squared dye-to-dye distance are indicated as vertical lines. Source data are provided as a Source Data file.

dA_{19}	5’-5ThioMC6-D/AAAAAAAAAAAAAAAAAAA/3AmMO/-3’
dC_{19}	5’-5ThioMC6-D/CCCCCCCCCCCCCCCCCCC/3AmMO/-3’
dT_{19}	5’-5ThioMC6-D/TTTTTTTTTTTTTTTTTTT/3AmMO/-3’
dT_{19}^{ab}	5’-5ThioMC6-D/TidSpTidSpTidSpTidSpTidSpTidSpTidSpTidSpTidSpT/3AmMO/-3’
dT_{38}	5’-5ThioMC6-D/TTTTTTTTTTTTTTTTTTTTTTTTTTTTTTTTTTTTTT /3AmMO/-3’
rA_{19}	5’-5ThioMC6-D/rArArArArArArArArArArArArArArArArArArA/3AmMO/-3’
rC_{19}	5’-5ThioMC6-D/rCrCrCrCrCrCrCrCrCrCrCrCrCrCrCrCrCrCrC/3AmMO/-3’
rU_{19}	5’-5ThioMC6-D/rUrUrUrUrUrUrUrUrUrUrUrUrUrUrUrUrUrUrU/3AmMO/-3’

Supplementary Table 1. Sequences of oligoribonucleotides (rA_{19}, rC_{19}, rU_{19}) and oligodeoxyribonucleotides (dA_{19}, dC_{19}, dT_{19}, dT_{38}, dT_{19}^{ab}).

5’-5ThioMC6-D/ dithiol with a 6-carbon spacer for maleimide labeling

/idSp/ 1,2’-Dideoxyribose modification used to insert single base space

/3AmMO/-3’ amino modifier for succinimidyl ester labeling

	$\tau_{fl(A488)}$ (ns)	$\tau_{fl(A594)}$ (ns)	$\tau_{rot(A488)}(\mathrm{ns})\|\alpha$ (%)	$\tau_{rot(A594)}(\mathrm{ns})\|\alpha$ (%)	τ_M(ns)
$\mathrm{dA_{19}}$	3.5	4.0	0.46 | 89	0.57 |76	1.55
$\mathrm{dC_{19}}$	3.9	4.0	0.40 | 87	0.65 |79	1.97
$\mathrm{dT_{19}}$	4.0	4.1	0.44 | 79	0.59 | 86	1.38
$\mathrm{rA_{19}}$	3.7	3.9	0.41 | 88	0.62 | 72	2.01
$\mathrm{rC_{19}}$	3.9	4.0	0.39 | 69	0.51 | 80	1.42
$\mathrm{rU_{19}}$	4.0	4.0	0.43 | 82	0.69 | 77	1.56

Supplementary Table 2. Results from polarization-resolved fluorescence lifetime analysis. To exclude the influence of the FRET process on the fluorescence lifetime and anisotropy decay analysis of the donor, we measured donor fluorescence decays of ssNAs labeled only with the donor. Note the reduction in fluorescence lifetime of the donor in the presence of purine bases ($\mathrm{dA_{19}}$ and $\mathrm{rA_{19}}$), indicating slight dynamic quenching of Alexa Fluor 488 by adenine. For $\mathrm{dT_{38}}$ and $\mathrm{dT_{19}^{ab}}$, we used the lifetimes measured for $\mathrm{dT_{19}}$.

	R_{HCG} (nm)	R_{GC} (nm)	R_{WLC} | l_p (nm)	$R_{SAW\nu}$ (nm) | ν	l_c (nm)	b (nm) | n
dA_{19}	6.4	7.3	6.8 | 1.3	6.7 | 0.71	19.1	0.55 | 34
dC_{19}	5.6	6.1	5.9 | 0.9	5.8 | 0.67	19.1	0.55 | 34
dT_{19}	5.5	5.8	5.6 | 0.9	5.6 | 0.66	19.1	0.55 | 34
dT_{19}^{ab}	5.3	6.1	5.9 | 1.1	5.8 | 0.67	19.1	0.55 | 34
dT_{38}	10.2	10.9	10.2 | 1.4	9.1 | 0.69	32.4	0.55 | 59
rA_{19}	5.5	6.1	5.9 | 0.9	5.8 | 0.67	19.1	0.55 | 34
rC_{19}	6	6.7	6.4 | 1.1	6.2 | 0.69	19.1	0.55 | 34
rU_{19}	5.1	5.6	5.5 | 0.8	5.4 | 0.65	19.1	0.55 | 34

Supplementary Table 3. Fit results (R, l_p, ν) obtained by relating the experimentally determined transfer efficiencies to the analytical polymer models and the values used for l_c, b, and n. The contour length, l_c, used for the analysis of the WLC model was calculated assuming an inter-phosphate distance of 0.7 nm for both ssRNA and ssDNA[5]. The linkers including donor and acceptor dye were modeled in PyMol[6] in an all-trans conformation, resulting in a total length of 5.8 nm for all constructs. For the SAW-ν polymer model, a segment length of *b* = 0.55 nm was assumed[7].

	dA_{19}	dC_{19}	dT_{19}	dT_{19}^{ab}	rA_{19}	rC_{19}	rU_{19}	dT_{38}
τ_{cd} (ns)	14 ±2	12 ±2	10 ±1	8 ±1	17 ±3	10 ±2	10 ±2	21 ±3
τ_r^{GC} (ns)	17 ±2	13 ±2	11 ±2	9 ±1	19 ±2	12 ±2	11 ±2	37±5
τ_r^{WLC} (ns)	15 ±2	12 ±2	10 ±1	8 ±1	18 ±3	11±2	10±2	29±4
$\tau_r^{SAW\nu}$ (ns)	15 ±2	12 ±2	10 ±1	8 ±1	17 ±3	10 ±2	10 ±2	21 ±3
τ_r^{HCG} (ns)	14 ±2	12 ±2	10 ±1	8 ±1	17 ±3	10 ±2	10 ±2	26 ±4
$\tau_r^{HCG_{BioEn}}$ (ns)	15 ±2	13 ±2	11 ± 1	9 ±1	17 ±3	10 ±2	11 ±2	31 ± 4
$\tau_r^{PM_{BioEn}}$ (ns)	16 ±2	13 ±2	11 ± 1	9 ±1	18 ±4	11 ±2	11 ±2	30 ± 4

Supplementary Table 4. Measured fluorescence correlation times, τ_{cd}, and resulting chain reconfiguration times, τ_r, obtained using Eq. 14 for the different polymer models (GC, WLC, SAW-v) and the HCG ensembles as well as for the reweighted distributions (HCG_{BioEn}, PM_{BioEn}) of ssDNAs and ssRNAs (150mM NaCl in HEPES buffer pH7). The uncertainties of τ_{cd} and τ_r represent the standard deviation from three independent measurements. Reconfiguration times in gray are only shown for completeness (see Bayesian ensemble refinement for details).

k_{ee} $(10^6\ s^{-1})$	dA_{19}	dC_{19}	dT_{19}	dT_{19}^{ab}	rA_{19}	rC_{19}	rU_{19}	dT_{38}
k_{ee}^{GC}	2.37	3.64	4.23	5.33	2.42	3.41	4.88	0.69
k_{ee}^{WLC}	1.48	2.39	2.80	3.51	1.56	2.20	3.27	0.53
k_{ee}^{SAWv}	0.76	1.26	1.52	1.86	0.84	1.13	1.76	0.33
$k_{ee}^{HCG_{BioEn}}$	0.27	0.26	0.41	0.39	0.26	0.21	0.40	0.04
$k_{ee}^{PM_{BioEn}}$	0.37	0.67	0.97	0.50	0.93	0.61	0.69	0.15

Supplementary Table 5. End-to-end contact rates (k_{ee}) obtained with Eq. 15 for the different polymer models (GC, WLC, SAW-*v*) and the HCG_{BioEn} ensembles.

	dA_{19}	dC_{19}	dT_{19}	dT_{19}^{ab}	rA_{19}	rC_{19}	rU_{19}	dT_{38}
$\langle E \rangle$	0.40±0.03	0.54±0.03	0.56±0.03	0.53±0.03	0.54±0.03	0.46±0.03	0.60±0.03	0.17±0.03
σ_ε^2	0.074±0.003	0.069±0.003	0.068±0.003	0.055±0.003	0.087±0.003	0.074±0.003	0.054±0.003	0.043±0.003

Supplementary Table 6. Experimentally determined variances, σ_ε^2, and mean transfer efficiencies, $\langle E \rangle$, of ssDNA and ssRNA (150mM NaCl in HEPES buffer pH7). Uncertainties of σ_ε^2 were estimated from the standard deviations of σ_ε^2 from relative donor and acceptor fluorescence lifetimes versus transfer efficiency. The uncertainty in $\langle E \rangle$ represents the systematic experimental uncertainty in transfer efficiency of ~0.03 in single-molecule FRET[8, 9].

Supplementary References

1. Antonik M., Felekyan S., Gaiduk A., Seidel C. A. Separating structural heterogeneities from stochastic variations in fluorescence resonance energy transfer distributions via photon distribution analysis. *J Phys Chem B* **110**, 6970-6978 (2006).
2. Nir E., Michalet X., Hamadani K. M., Laurence T. A., Neuhauser D., Kovchegov Y., Weiss S. Shot-noise limited single-molecule FRET histograms: comparison between theory and experiments. *J Phys Chem B* **110**, 22103-22124 (2006).
3. Kalinin S., Felekyan S., Valeri A., Seidel C. A. Characterizing multiple molecular states in single-molecule multiparameter fluorescence detection by probability distribution analysis. *J Phys Chem B* **112**, 8361-8374 (2008).
4. Abou Assi H., Garavís M., González C., Damha M. J. i-Motif DNA: structural features and significance to cell biology. *Nucleic Acids Res.* **46**, 8038-8056 (2018).
5. Ho P. S., Megan C. DNA Structure: Alphabet Soup for the Cellular Soul. In: *DNA Replication* (eds Herve S.). IntechOpen (2011).
6. DeLano W. L. The PyMOL molecular graphics system. *http://www.pymol.org* (2002).
7. Zheng W., Zerze G. H., Borgia A., Mittal J., Schuler B., Best R. B. Inferring properties of disordered chains from FRET transfer efficiencies. *J Chem Phys* **148**, 123329 (2018).
8. Holmstrom E. D., Holla A., Zheng W., Nettels D., Best R. B., Schuler B. Accurate Transfer Efficiencies, Distance Distributions, and Ensembles of Unfolded and Intrinsically Disordered Proteins From Single-Molecule FRET. *Methods Enzymol* **611**, 287-325 (2018).
9. Hellenkamp B., Schmid S., Doroshenko O., Opanasyuk O., Kuhnemuth R., Rezaei Adariani S., . . . Hugel T. Precision and accuracy of single-molecule FRET measurements-a multi-laboratory benchmark study. *Nat. Methods* **15**, 669-676 (2018).
